# Hepatic profile analyses of tipranavir in Phase II and III clinical trials

**DOI:** 10.1186/1471-2334-9-203

**Published:** 2009-12-14

**Authors:** Jaromir Mikl, Mark S Sulkowski, Yves Benhamou, Douglas Dieterich, Stanislas Pol, Jürgen Rockstroh, Patrick A Robinson, Mithun Ranga, Jerry O Stern

**Affiliations:** 1 SUNY at Albany, School of Public Health, Rensselaer NY, USA; 2 Boehringer Ingelheim Pharmaceuticals, Inc., Ridgefield, CT, USA; 3 Division of Infectious Diseases, Johns Hopkins University School of Medicine, Baltimore, USA; 4 Service d'Hépato-Gastroentérologie, Hôpital Pitié-Salpêtrière, Paris, France; 5 Mount Sinai School of Medicine, New York, New York, USA; 6 Hôpital Necker, APHP, Unité d'Hépatologie, Paris, France; 7 Department of Medicine I, University of Bonn, Bonn, Germany

## Abstract

**Background:**

The risk and course of serum transaminase elevations (TEs) and clinical hepatic serious adverse event (SAE) development in ritonavir-boosted tipranavir (TPV/r) 500/200 mg BID recipients, who also received additional combination antiretroviral treatment agents in clinical trials (TPV/r-based cART), was determined.

**Methods:**

Aggregated transaminase and hepatic SAE data through 96 weeks of TPV/r-based cART from five Phase IIb/III trials were analyzed. Patients were categorized by the presence or absence of underlying liver disease (+LD or -LD). Kaplan-Meier (K-M) probability estimates for time-to-first US National Institutes of Health, Division of AIDS (DAIDS) Grade 3/4 TE and clinical hepatic SAE were determined and clinical actions/outcomes evaluated. Risk factors for DAIDS Grade 3/4 TE were identified through multivariate Cox regression statistical modeling.

**Results:**

Grade 3/4 TEs occurred in 144/1299 (11.1%) patients; 123/144 (85%) of these were asymptomatic; 84% of these patients only temporarily interrupted treatment or continued, with transaminase levels returning to Grade ≤ 2. At 96 weeks of study treatment, the incidence of Grade 3/4 TEs was higher among the +LD (16.8%) than among the -LD (10.1%) patients. K-M analysis revealed an incremental risk for developing DAIDS Grade 3/4 TEs; risk was greatest through 24 weeks (6.1%), and decreasing thereafter (>24-48 weeks: 3.4%, >48 weeks-72 weeks: 2.0%, >72-96 weeks: 2.2%), and higher in +LD than -LD patients at each 24-week interval. Treatment with TPV/r, co-infection with hepatitis B and/or C, DAIDS grade >1 TE and CD4^+ ^> 200 cells/mm^3 ^at baseline were found to be independent risk factors for development of DAIDS Grade 3/4 TE; the hazard ratios (HR) were 2.8, 2.0, 2.1 and 1.5, respectively. Four of the 144 (2.7%) patients with Grade 3/4 TEs developed hepatic SAEs; overall, 14/1299 (1.1%) patients had hepatic SAEs including six with hepatic failure (0.5%). The K-M risk of developing hepatic SAEs through 96 weeks was 1.4%; highest risk was observed during the first 24 weeks and decreased thereafter; the risk was similar between +LD and -LD patients for the first 24 weeks (0.6% and 0.5%, respectively) and was higher for +LD patients, thereafter.

**Conclusion:**

Through 96 weeks of TPV/r-based cART, DAIDS Grade 3/4 TEs and hepatic SAEs occurred in approximately 11% and 1% of TPV/r patients, respectively; most (84%) had no significant clinical implications and were managed without permanent treatment discontinuation. Among the 14 patients with hepatic SAE, 6 experienced hepatic failure (0.5%); these patients had profound immunosuppression and the rate appears higher among hepatitis co-infected patients. The overall probability of experiencing a hepatic SAE in this patient cohort was 1.4% through 96 weeks of treatment. Independent risk factors for DAIDS Grade 3/4 TEs include TPV/r treatment, co-infection with hepatitis B and/or C, DAIDS grade >1 TE and CD4^+ ^> 200 cells/mm^3 ^at baseline.

**Trial registration:**

US-NIH Trial registration number: NCT00144170

## Background

The introduction of highly active antiretroviral therapy (HAART) is responsible for a substantial decline in AIDS progression, opportunistic infections and mortality in HIV-1-infected individuals in recent years [1-3]. However, in the presence of antiretroviral (ARV) drugs and non-adherence, resistant strains emerge and contribute to therapeutic failure [4]. Tipranavir (TPV; Aptivus^® ^is a next-generation protease inhibitor (PI), used in combination with low-dose ritonavir (RTV;/r) in combination with other background antiretroviral agents (TPV/r-based cART) for treatment-experienced, HIV-1 infected patients with resistance to more than one PI. TPV/r has shown sustained activity against viruses with up to 20 PI mutations [5]. However, TPV/r-based cART use is associated with a risk of elevated serum levels of alanine aminotransferase (ALT) and/or aspartate aminotransferase (AST) transaminases [6].

In general, HAART may be associated with elevated serum transaminases [7]. The reported incidence of severe transaminase elevations associated with HAART varies from a low of 2% to as high as 18% [8]. However, reports from other investigators indicate that as many as 6%-30% of ARV treated patients, particularly those receiving protease inhibitors, may experience significant increases in serum liver enzymes and consequently may have to discontinue treatment [9,10]. Patients co-infected with viral hepatitis are at increased risk for hepatotoxicity [8,11-13]. Considering that between 10% - 50% of HIV infected persons in the US and Europe are estimated to be co-infected with HCV [14-17] hepatotoxicity becomes a challenge when treating these patients. Severe hepatotoxicity occurs in up to 6% of patients on HAART [7].

A recent study evaluated patients receiving PI-based ARV therapy, with or without RTV co-administration [9]; the risk of developing hepatotoxicity was similar across the PIs evaluated and a boosting dose of RTV was associated with a much lower risk than was observed with therapeutic RTV dosing [9]. The study concluded that, while drug-induced liver toxicity was an important consideration for HIV treatment decisions, potential efficacy and pre-existing resistance primarily influence the selection of a cART regimen [9]. In a recent report, the Kaplan-Meier (K-M) risk estimates of US National Institutes of Health, Division of AIDS (DAIDS) [18] Grade 3/4 ALT elevations were 9.9% and 2.8%, respectively, in patients treated with TPV/r or RTV-boosted comparator PIs (CPI/r) in the large Phase III RESIST trials at 48 weeks [19]. Multivariate analyses in TPV/r-based cART patients indicated that the most significant risk factors for Grade 3/4 ALT/AST levels in the TPV/r and CPI/r groups were elevated baseline transaminases and hepatitis co-infection [19]. Others observed similar results, identifying elevated transaminases at baseline and/or hepatitis co-infection as risk factors associated with liver toxicity among HIV infected patients treated with antiretrovirals [20-24].

The current analysis is based largely on patients enrolled in the RESIST trial, which demonstrated superior TPV/r efficacy and durability in suppressing viral load (VL), compared to CPI/r in more than 1400 patients with PI resistance, advanced HIV infection and few, if any, other treatment options [6].

The objective of this hepatic safety analysis was to evaluate the risk and natural history of serum ALT/AST transaminase elevations and clinical hepatic serious adverse event (SAE) development among treatment-experienced patients receiving the approved standard dose of 500/200 mg BID TPV/r in Phase IIb and III trials through 96 weeks of treatment. The development of DAIDS Grade 3/4 ALT/AST elevations was further evaluated in patients categorized as having underlying liver disease (+LD; baseline transaminase elevations DAIDS Grade >1 [18] and/or hepatitis B/C virus [HBV/HCV] co-infection) or no underlying liver disease (-LD; baseline transaminase elevations Grade ≤ 1 and no HBV/HCV co-infection).

## Methods

Aggregated safety data were pooled from the five Phase IIb and III trials which comprised the clinical development program for TPV/r. Patients in these trials were highly treatment-experienced and received the approved dose of TPV/r 500/200 mg BID. The five trials in which these patients were enrolled were two Phase II trials (1182.51 [N = 67], 1182.52 [n = 72]) [25,26], two pivotal Phase III trials (1182.12 [RESIST 1, n = 311], 1182.48 [RESIST 2, n = 436]) [6,27,28] and one "roll-over" trial (1182.17 [n = 413]) [29] that allowed patients from completed Phase II trials to continue to receive TPV/r 500/200 mg BID and provided TPV/r to those in RESIST who failed on the CPI/r therapy. The RESIST-1 and RESIST-2 trials were open-label, randomized trials that compared TPV/r with CPI/r regimens in 21 countries in Europe, North and South America, and Australia [6,27,28]. Trial 1182.52 was a randomized, dose-selection trial thatcompared three dose regimens of TPV/r, including the dose of 500/200 mg BID [25]. Trial 1182.51 was a randomizedpharmacokinetic and safety comparison of TPV/r versus CPI/r for 2 weeks, followed by a dual-boosted regimen in patients who were ineligible for the pivotal Phase III trials because of resistance requirements [26].

All patients included in these five trials were HIV-1-infected male and female adults (aged ≥ 18 years) with ≥ 3 months of prior treatment with three classes of ARVs (nucleoside reverse transcriptase inhibitors [NRTIs], non-nucleoside reverse transcriptase inhibitors [NNRTIs], and PIs), including at least two prior PI-based regimens (one being the regimen at screening), who had a VL of ≥ 1000 copies/mL of plasma HIV-1 RNA at baseline. Patients with HBV or HCV were eligible for inclusion if serum ALT/AST transaminase levels were DAIDS Grade 2 (≤2.5 × the upper limit of normal [ULN] reference range) or less [18]. Patients who were failing CPI/r in the RESIST trials or who were treated with TPV/r in Phase II trials were allowed to participate in the open-label TPV/r roll-over study (1182.17) and were included in the hepatic safety analyses.

All studies were designed and monitored in accordance with the International Conference on Harmonization guidance for Good Clinical Practice (ICH GCP) and written informed consent was obtained from all study participants. All study protocols, informed consent forms and trial documentation was reviewed and approved by the institutional review boards/local ethics committees of participating centers.

The course of transaminase elevations and hepatic SAEs through 96 weeks was characterized. Transaminase elevations were categorized according to NIH, DAIDS [18] system for Grading the Severity of Adult and Pediatric Adverse Events based on multiples of the Upper Limit of Normal (ULN) reference range for ALT/AST: DAIDS Grade 0 defined as ALT/AST within normal range through < 1.25 × ULN; Grade 1 defined as mild elevation (1.25-2.5 × ULN); DAIDS Grade 2 defined as moderate elevation (>2.5-5.0 × ULN); DAIDS Grade 3 defined as ALT/AST > 5-10 × ULN; and DAIDS Grade 4 defined as ALT/AST >10 × ULN. K-M probability estimates for time-to-first DAIDS Grade 3/4 ALT/AST elevation and time-to-first clinical hepatobiliary SAE were determined. Clinical actions and outcomes following the development of DAIDS Grade 3/4 transaminase elevation were analyzed with regards to treatment continuation, interruption or discontinuation of TPV/r.

Potential risk factors for DAIDS Grade 3/4 for ALT and/or AST were assessed through multivariate Cox regression statistical modeling and included all RESIST 1 and 2 patients (N = 1486) in order to compare TPV/r and CPI/r treated patients.

Hepatobiliary adverse events (AEs) were defined as any AE with a MedDRA (ver. 8.1) preferred term falling into hepatobiliary system organ class (SOC) and included serious and non-serious hepatobiliary AEs regardless of severity. All of these events were considered to represent "symptomatic" AEs. Complete clinical profiles (all laboratory and reported AEs from baseline throughout the participation of the patient in the study) were created and underwent medical review. Events were excluded as representing symptomatic "hepatic" events if they were due to biliary disease alone (e.g. cholecystitis, gallbladder cyst, choledocholithiasis), hepatic neoplasm alone (primary or secondary), or if they represented only asymptomatic elevations of liver laboratory tests, with the absence of any other associated clinical AEs (e.g., fever, malaise, nausea, abdominal pain, etc).

Patients were classified as +LD retrospectively by the presence of either chronic viral hepatitis and/or elevated serum transaminases DAIDS Grade >1 at baseline [19]. Study protocols excluded patients with screening ALT/AST elevation of Grade ≥ 2; however, some patients (n = 48) who met the screening criteria were found to have DAIDS Grade 2 elevations at baseline on the first study visit and were included in the trials. Chronic HCV infection was defined by the detection of HCV RNA. Chronic HBV infection was assessed at baseline by the presence of hepatitis B surface antigen (HBV DNA was not measured). Patients who were not co-infected with HBV or HCV and who had baseline transaminase elevations of Grade 1 or less were classified as -LD for these analyses.

## Results

### Baseline characteristics

Baseline characteristics of all study patients are presented in Table 1. Data aggregated from the five TPV/r trials identified 1299 treatment-experienced patients who received the approved 500/200 mg BID dose of TPV/r. Of these, 1088 were classified as -LD patients and 179 as +LD patients. The remaining 32 patients had either HBV/HCV co-infection status or transaminase elevation data missing at baseline and therefore are listed as unclassified and were excluded from the +LD and -LD sub-analyses.

**Table 1:** Demographic and baseline characteristics of TPV/r 500/200 mg patients in trials 1182.12, 1182.48, 1182.51, 1182.52 and 1182.17 by risk

	All TPV/r^1^ N = 1299	TPV/r -LD^2^ N = 1088	TPV/r +LD^2^ N = 179	TPV/r SAE N = 14
Age (years):				
Median	43	43	42	42
Range	17-80	17-80	18-72	35-63

Gender [N (%)]:				
Male	1124 (86.5)	939 (86.3)	160 (89.4)	13 (92.9)
Female	175 (13.5)	149 (13.7)	19 (10.6)	1 (7.1)

Baseline HIV RNA (log_10 _copies/mL):				
Median	4.8	4.8	4.7	4.9
Range	1.7-6.5	1.7-6.5	2.7-6.3	3.6-5.6

Baseline CD4+ cell count (cells/mm^3^):				
Median	156	158	151	70
Range	1-1893	1-1893	1-702	4-337

Hepatitis co-infection [N (%)]:				
HBsAg-/HCV RNA-	1136 (87.5)	1088 (100.0)	37 (20.7)	9 (64.3)
HBsAg+	55 (4.2)	0 (0.0)	55 (30.7)	2 (14.3)
HCV RNA+	83 (6.4)	0 (0.0)	83 (46.4)	3 (21.4)
HBsAg+/HCV RNA+	2 (0.2)	0 (0.0)	2 (1.1)	0 (0.0)
missing	23 (1.8)	0 (0.0)	2 (1.1)	0 (0.0)

Baseline DAIDS Grade ≥2 ALT/AST^3^	51 (3.9)	0 (0.0)	51 (28.5)	0 (0.0)

^1^Thirty-two patients had missing information and were unable to be classified into one of the two risk groups. These patients were more likely to be female (21.9%) with lower CD4+ counts at baseline (median 121 cells/mm^3^).

^2^+LD: patients with underlying liver disease (baseline evidence of active HBV/HCV infection or ALT/AST Grade > 1); -LD: patients with no apparent liver disease (absence of active HBV/HCV infection and ALT/AST Grade ≤ 1).

^3^At baseline, 48 patients had DAIDS Grade 2 ALT/AST, while three patients had DAIDS Grade 3 ALT/AST just prior to first dose of TPV/r.

ALT = alanine aminotransferase; AST = aspartate aminotransferase; DAIDS = Division of AIDS; HBsAg = hepatitis B surface antigen; HBV = hepatitis B virus; HCV = hepatitis C virus; TPV/r = ritonavir-boosted tipranavir 500/200 mg BID

The patients were mostly males (86.5%) and there was similar gender distribution between the risk groups (Table 1). The median baseline HIV-1 RNA and CD4+ cell counts were similar between the two risk groups; -LD patients had a median HIV-1 RNA level of 4.8 log_10 _copies/mL and a median CD4+ cell count of 158 cells/mm^3 ^at baseline, +LD patients had a median HIV RNA of 4.7 log_10 _copies/mL and a median CD4+ cell count of 151 cells/mm^3 ^at baseline.

Compared to patients who did not experience hepatic SAEs, the 14 patients who experienced hepatic SAE were slightly older, and presented with higher HIV RNA counts and lower CD4^+ ^counts at baseline (Table 1). All but one of these patients responded to TPV/r treatment: the median maximal reduction from baseline in HIV RNA count was 5.3 log_10 _and the median maximal CD4+ count increase from baseline was 74.5 cells/mm^3 ^(data not shown).

### DAIDS Grade 3/4 ALT and/or AST transaminase elevations

In the aggregate Phase II and III TPV/r trials (n = 1299), 11.1% of TPV/r patients receiving 500/200 mg BID developed DAIDS Grade 3/4 ALT/AST elevations through 96 weeks of TPV/r-based cART treatment in the studies. The incidence of DAIDS Grade 3/4 events was 11.1% (144/1299 subjects); of these 6.9% were Grade 3 and 4.2% were Grade 4 elevations.

**Figure 1:**
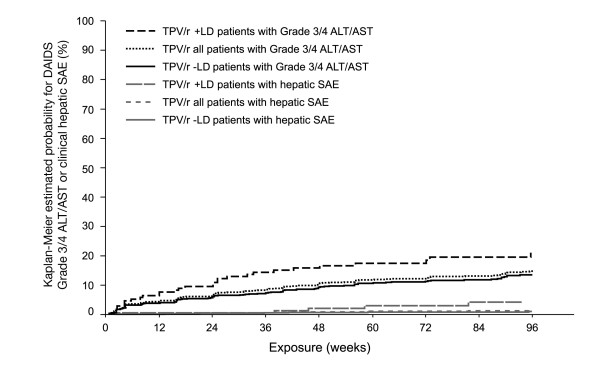
**Kaplan-Meier estimates for time-to-first Grade 3/4 ALT/AST elevations and to first hepatobiliary serious adverse events (SAEs) among TPV/r recipients by baseline risk group**. Log-rank test p-value comparing -LD vs. +LD:. Incidence of Hepatic SAEs p = 0.0125. Incidence of DAIDS Grade 3/4 ALT/AST p = 0.0072. TPV/r +LD patients with Grade 3/4 ALT/AST = TPV/r patients co-infected with HBV/HCV or with baseline ALT/AST DAIDS >1, time-to-first DAIDS ὅ3 ALT/AST. TPV/r -LD patients with Grade 3/4 ALT/AST = TPV/r patients not co-infected with HBV/HCV and with baseline ALT/AST DAIDS ὄ1, time-to-first DAIDS ὅ3 ALT/AST. TPV/r +LD patients with hepatic SAE = TPV/r patients co-infected with HBV/HCV or with baseline ALT/AST DAIDS >1, time-to-first onset date of hepatic SAE. TPV/r -LD patients with hepatic SAE = TPV/r patients not co-infected with HBV/HCV and with baseline ALT/AST DAIDS ὄ1, time-to-first onset date of hepatic SAE. +LD = patients with underlying liver disease (baseline evidence of active HBV/HCV infection or ALT/AST DAIDS >1); -LD = patients with no apparent liver disease (absence of active HBV/HCV infection and ALT/AST Grade ≤ 1); ALT = alanine aminotransferase; AST = aspartate aminotransferase; DAIDS = Division of AIDS; HBV = hepatitis B virus; HCV = hepatitis C virus; TPV/r = ritonavir-boosted tipranavir.

Cumulative K-M estimates through 96 weeks of study treatment for time-to-first DAIDS Grade 3/4 ALT/AST elevations are presented in Table 2 and Figure 1. In the overall patient population, the cumulative risk of developing a DAIDS Grade 3/4 ALT/AST elevation while on study treatment was greatest in the first 24 weeks (6.1%) compared to subsequent 24 week periods. The subsequent cumulative risk estimate was 9.5% at 48 weeks (an accumulation of 3.4% additional risk in the period from 24 to 48 weeks). At 72 weeks the cumulative risk was 11.5% (additional 2.0% in the period from 48 weeks to 72 weeks) and at 96 weeks the cumulative risk was 13.7% (an additional 2.2%, for 72 to 96 weeks). The proportion of patients with DAIDS Grade 3/4 transaminase elevations by Week 96 was lower among -LD patients (10.1%; 110/1088), compared to +LD patients (16.8%; 30/179). The K-M cumulative risk for Grade 3/4 ALT/AST elevations was nearly 2-fold higher in +LD patients compared to their -LD counterparts at each 24 week interval (Table 2).

**Table 2:** Kaplan-Meier estimates for time-to-first DAIDS Grade 3/4 ALT and/or AST elevation and time-to-first hepatic serious adverse event (SAE) among TPV/r 500/200 mg patients in trials 1182.12, 1182.48, 1182.51, 1182.52 and 1182.17

96 week CR(%)^1^	DAIDS^2 ^Grade 3/4 ALT/AST			Hepatic SAE		
	**TPV/r** **All** **N = 1299**	**TPV/r-LD^3^** **N = 1088**	**TPV/r +LD^3^** **N = 179**	**TPV/r** **All** **N = 1299**	**TPV/r-LD^4^** **N = 1088**	**TPV/r+LD^4^** **N = 179**

Week 24	6.1	5.4	9.4	0.5	0.5	0.6

Week 48	9.5	8.3	15.6	0.9	0.7	2.1

Week 72	11.5	10.3	17.2	1.1	0.8	3.0

Week 96	13.7	12.5	20.6	1.4	1.0	4.3

^1^Cumulative rate through 96 weeks.

^2^DAIDS Grade 3 is defined as ALT/AST = 5.1 to 10 × ULN reference range. DAIDS Grade 4 is defined as ALT/AST >10 × ULN reference range.

^3^Log-rank test comparing -LD versus +LD patients: p < 0.05.

^4^Log-rank test comparing -LD versus +LD patients: p < 0.05.

+LD = patients with underlying liver disease (baseline evidence of active HBV/HCV infection or ALT/AST Grade >1); -LD = patients with no apparent liver disease (absence of active HBV/HCV infection and ALT/AST Grade ≤ 1); ALT = alanine aminotransferase; AST = aspartate aminotransferase; DAIDS = Division of AIDS; HBV = hepatitis B virus; HCV = hepatitis C virus; SAE = serious adverse event; TPV/r = ritonavir-boosted tipranavir; ULN = upper limit of normal

### Risk factors for DAIDS Grade 3/4 TE

The RESIST 1 and 2 trials were randomized controlled trials that compared responses between TPV/r- based and comparator PI/r- (CPI/r-) based cART. The design and the patient population have been described previously [19,27,28,30,31]. To further understand the association of risk factors with the development of severe liver transaminase elevations, Cox regression model was constructed. This model examined time to first DAIDS Grade 3/4 ALT and/or AST elevations in RESIST patients, using 96+ week data. Risk factors found to be associated with time to first Grade 3/4 ALT and/or AST elevations while controlling for other factors in the model (see Table 3) included TPV/r treatment (HR: 2.8; p < 0.05), HBV/HCV co-infection (HR: 2.0; p < 0.05), elevated (DAIDS Grade ≥2) ALT and/or AST at baseline (HR: 2.1; p < 0.10), and baseline CD4 >200 cells/mm^3 ^(HR: 1.5; p < 0.10).

**Table 3:** Cox regression for time to first occurrence of DAIDS Grade 3 or 4 ALT and/or AST abnormalities in TPV/r-based and comparator PI/r- (CPI/r-) based cART RESIST patients

Parameter	HR^1 ^(p-value)	95% Confidence Interval
Treatment group:	2.77 (<0.0001)	1.71, 4.48
TPV/r v CPI/r		

HBV or HCV co-infection:	2.00 (0.0057)	1.22, 3.27
yes vs. no		

ALT/AST at baseline:	2.05 (0.0709)	0.94, 4.48
Grade ≥2 vs. ≤Grade 1		

CD4+ cells at baseline:	1.46 (0.0643)	0.98, 2.18
>200 vs. ≤200 cells/mm^3^		

^1 ^Hazard Ratio

Model further controlled for sex, race and concurrent use of T-20 (none were statistically significant).

Two additional Cox models examined only the TPV/r patients in RESIST 1 and 2, and compared the risk of DAIDS Grade 3/4 ALT and/or AST elevation in patients who were either HBV/HCV co-infected or who had DAIDS Grade ≥2 for ALT and/or AST at baseline versus those patients who had neither co-infection nor baseline ALT and/or AST elevation. The analyses used 96+ week data. In both comparisons, the risk was significantly elevated (HR: 1.8, p < 0.05) in patients who had co-infection or ≥2 DAIDS Grade ALT and/or AST at baseline (Data not shown).

### Clinical outcomes following Grade 3/4 ALT/AST transaminase elevations

Of the 144 patients who developed DAIDS Grade 3/4 ALT/AST elevations by Week 96, there were 123 (85%) who had no associated symptomatic hepatic events (asymptomatic). Over this period, 1.6% (21/1299) of TPV/r 500/200 mg BID recipients developed DAIDS Grade 3/4 ALT/AST elevations, accompanied by symptomatic hepatic AEs (serious and/or non-serious) and 0.3% (n = 4) of all TPV/r patients developed a hepatic SAE in association with a DAIDS Grade 3/4 ALT/AST elevation.

**Figure 2:**
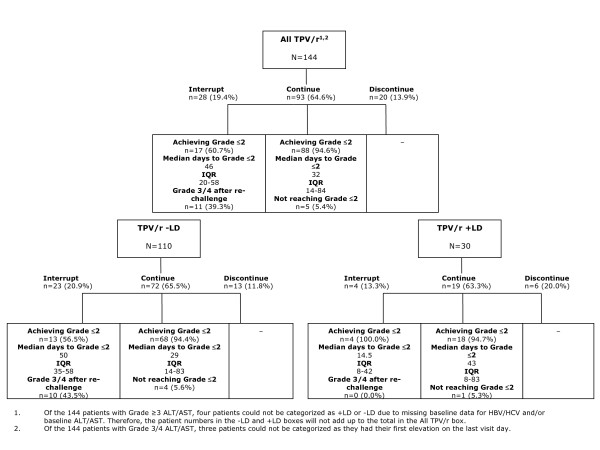
**Actions and outcomes of Grade 3/4 ALT/AST elevations among TPV/r recipients**. TPV/r +LD patients = TPV/r patients co-infected with HBV/HCV or with baseline ALT/AST DAIDS >1. TPV/r -LD patients = TPV/r patients not co-infected with HBV/HCV and with baseline ALT/AST DAIDS ὄ1. +LD = patients with underlying liver disease (baseline evidence of active HBV/HCV infection or ALT/AST DAIDS >1); -LD = patients with no apparent liver disease (absence of active HBV/HCV infection and ALT/AST Grade ≤ 1); ALT = alanine aminotransferase; AST = aspartate aminotransferase; HBV = hepatitis B virus; HCV = hepatitis C virus; IQR = interquartile range; TPV/r = ritonavir-boosted tipranavir.

The majority (84.0%) of the 144 patients were either able to continue TPV/r treatment or re-introduce treatment following interruption (Figure 2).

Following the development of DAIDS Grade 3/4 ALT/AST elevations, 64.6% (93/144) of patients continued TPV/r treatment without interruption, and in 94.6% of these, ALT/AST returned to Grade 2 or less in a median of 32 days (IQR = 14-84 days), while remaining on treatment.

There were 28/144 (19.4%) patients who had at least one treatment interruption. For 17/28 (60.7%) patients who interrupted therapy, elevated transaminases returned to DAIDS Grade 2 or less after a median of 46 days (IQR = 20-58 days). However, 11/28 (39.3%) patients who had treatment interruption developed subsequent recurrence(s) of DAIDS Grade 3/4 transaminases that led to discontinuation.

Twenty of the 144 (13.9%) patients with DAIDS Grade 3/4 ALT/AST elevations discontinued treatment at the first Grade 3/4 elevation. Three patients developed their first severe transaminase elevations on the last visit for which follow-up data were available.

The course of action taken among -LD and +LD TPV/r patients was similar (Figure 2), with the majority (81/110 [74%] -LD patients; 22/30 [73%] +LD patients) able to continue, or interrupt and re-introduce, TPV/r treatment with a subsequent decrease in transaminase elevations to DAIDS Grade 2 or less. All -LD patients who continued with TPV/r treatment had transaminase levels return to DAIDS Grade 2 or less after a median of 29 days. Similarly, among +LD patients who continued with TPV/r treatment, all but one patient had transaminase levels decrease to DAIDS Grade 2 or less, although the median recovery time was longer than in -LD patients (43 days).

### Clinical hepatic serious adverse events

Among the 1299 treatment-experienced TPV/r patients, 14 (1.1%) patients experienced hepatic (non-biliary and non-laboratory) SAEs.

The cumulative risk of clinical hepatic SAEs remained low through 96 weeks (0.5% at Week 24, 0.9% at Week 48, and 1.4% at Week 96) (Table 2 and Figure 1). Among -LD and +LD patients, the cumulative risks of hepatic SAEs were similar during the first 24 weeks (0.5% and 0.6%, respectively). However, after Week 24, the risks of clinical hepatic SAEs increased disproportionately among +LD patients compared to -LD patients. K-M rates of +LD patients (Figure 1) increased to 2.1% at Week 48 (an accumulation of 1.5% additional risk from Week 24 to Week 48), to 3.0% at Week 72 (accumulated additional risk of 0.9% from Week 48 to 72), and to 4.3% at Week 96 (accumulated additional risk of 1.3% from Week 72 to 96). For -LD patients, the corresponding accumulation of additional risk was 0.2% from Week 24 to Week 48, 0.1% from Week 48 to 72, and 0.2% from Week 72 to 96.

Among the +LD patients there were 5/179 (2.8%) with hepatic SAEs; two with hepatic failure, one with hepatic steatosis, one with hepatocellular damage and one with isolated hyperbilirubinemia. All five were co-infected with HBV or HCV. There were 9/1088 -LD patients (0.8%) with hepatic SAEs; four with hepatic failure, two with hepatotoxicity or toxic hepatitis, and one patient each with steatosis, cirrhosis, hepatosplenomegaly.

Among all 1299 TPV/r-treated patients, six (0.5%) had a hepatic SAE denoting hepatic failure. Five had fatal outcomes. These events occurred in patients with concomitant end-stage AIDS events, including disseminated *Mycobacterium avium intracellulare*, visceral leishmaniasis, Burkitt's lymphoma, squamous carcinoma, sepsis and multi-organ failure. The sixth patient recovered rapidly and was discharged from the hospital after five days, suggesting the diagnosis may have not been entirely accurate. An apparent higher risk among hepatitis co-infected patients was observed; two of the six hepatic failure cases occurred among hepatitis co-infected patients (1.4%) while four occurred among non-hepatitis co-infected patients (0.4%; data not shown). In all but one case, the extent of the role of TPV in the development of liver failure is uncertain.

The occurrence of Grade 3/4 ALT/AST elevations and clinical hepatic SAEs among TPV/r 500/200 mg BID patients who were -LD and +LD is presented in Table 4. Of the 144 patients who developed Grade 3/4 ALT/AST elevations, four subsequently developed a hepatic SAE through 96 weeks, suggesting that transaminase elevations may be a poor predictor of clinical hepatic SAEs (positive predictive value [PPV]: 2.8%). The +LD patients had a PPV of just 3.3%, with only 1/30 patients with Grade 3/4 ALT/AST elevations developing a hepatic SAE. The -LD patients had a PPV of 2.7%, with 3/110 patients with a Grade 3/4 ALT/AST elevation through 96 weeks developing a hepatic SAE.

**Table 4:** Occurrence of DAIDS Grade 3/4 ALT/AST and hepatic serious adverse events (SAEs) among -LD and +LD TPV/r 500/200 mg BID patients

	-LD patients^1^		+LD patients^1^	
**ALT/AST DAIDS >2^2^**	**Hepatic SAE**		**Hepatic SAE**	

	**Yes**	**No**	**Yes**	**No**

Yes	3	107	1	29

No	6	970	4	141

^1^Thirty-two patients had missing information and were unable to be classified into one of the two risk groups; none of these patients experienced a hepatic SAE.

+LD = patients with underlying liver disease (baseline evidence of active HBV/HCV infection or ALT/AST Grade >1); -LD = patients with no apparent liver disease (absence of active HBV/HCV infection and ALT/AST Grade ≤ 1); ALT = alanine aminotransferase; AST = aspartate aminotransferase; DAIDS = Division of AIDS; HBV = hepatitis B virus; HCV = hepatitis C virus; TPV/r = ritonavir-boosted tipranavir

^2^ALT/AST on treatment DAIDS Grade 3/4

Among -LD and +LD patients treated with TPV/r 500/200 mg BID in the five trials comprising the TPV/r development program, monitoring of transaminase elevations to determine which patients may be at risk for clinical hepatic SAEs appears to have limited value, with corresponding sensitivity values of 33.3% and 20.0%. In both patient groups, regardless of the development of Grade 3/4 ALT/AST elevation, the large majority of patients did not experience a clinical hepatic SAE.

## Discussion and Conclusions

The goal of cART is to achieve durable HIV suppression and sustained improvements in immune function by the delivery of an ARV regimen that also provides adequate safety and tolerability. Adverse events are associated with all ARV agents and may influence decisions to switch or discontinue therapy. Since some individuals may be predisposed to ARV therapy-associated AEs, safety considerations should take into account underlying conditions, concomitant medications and history of drug intolerance [11]. All ARV drugs have been associated with development of Grade 3/4 ALT/AST elevations [11,32]; and these events are more frequent in patients with HBV/HCV co-infection or increased baseline hepatic transaminase levels [11,32-34]. However, the cause of liver enzyme elevations is often unclear, particularly among HIV-infected individuals with advanced disease. Further, in some asymptomatic individuals, elevated transaminases may resolve spontaneously despite continued drug treatment [35]. This may reflect an adaptive host response as seen with some drugs causing hepatotoxicity [36,37].

The development of DAIDS Grade 3/4 transaminase elevations and clinical hepatic SAEs in treatment-experienced HIV-positive patients receiving ARV therapy with TPV/r 500/200 mg BID varies according to baseline factors that define -LD and +LD patients. The K-M cumulative risk estimate of patients with DAIDS Grade 3/4 elevations by Week 96 was lower among the -LD patients (12.5%), compared to the +LD patients (20.6%).

The large majority of TPV/r patients who developed DAIDS Grade 3/4 ALT/AST elevations remained asymptomatic and did not develop clinical signs or symptoms of liver-related SAEs. Among -LD patients, the cumulative risk of experiencing a hepatic SAE was low (1.0%) through 96 weeks of treatment, with most occurring in the first 6 months. As expected, +LD patients tended to have a greater risk of developing a hepatic SAE than -LD patients.

Per protocol, any patient experiencing DAIDS 4 toxicity, according to the GCP and ICH guidelines, was to be discontinued from treatment. However, the patient population consisted of patients with advanced stage of HIV disease, presenting with opportunistic infections and the TPV/r was their last option for treatment. These compassionate trial designs were developed and approved by the regulatory authorities and key opinion leaders and the continued treatment despite toxicity was a decision between the principal investigator and the patient on a case by case basis with very close patient monitoring. Consequently, many patients who developed DAIDS Grade 3/4 ALT/AST elevations on TPV/r therapy were able to continue treatment uninterrupted or resume treatment after temporary discontinuation, with transaminase levels returning to DAIDS Grade 2 or less during the treatment period. Interestingly, changes in serum ALT/AST transaminase levels associated with TPV/r treatment were not typically predictive of the development of clinical hepatic SAEs. This lack of association of serum ALT/AST levels and clinical events has been observed with other medications. For example, at an FDA-AASLD-PhRMA (Food and Drug Administration-American Association for the Study of Liver Diseases-Pharmaceutical Research and Manufacturers of America) meeting on drug hepatotoxicity [36], it was pointed out that some drugs, such as isoniazid, are associated with a high risk of substantial transaminase elevations. However, these transaminase elevations are not strongly predictive of clinical hepatic events (low PPV) and clinical monitoring has become increasingly more important than laboratory monitoring. Nevertheless, transaminase elevations are a marker for hepatocellular liver injury so, despite poor PPV, increased transaminase monitoring is likely to be of value in +LD patients in whom the clinical significance of hepatic injury may be greater.

Multivariate analyses revealed hepatitis co-infection and baseline liver impairment being independently associated with increased risk of severe liver transaminase elevations. Others have also identified chronic viral hepatitis and abnormal baseline liver enzyme levels as risk factors for increased risk of hepatotoxicity with other ARV regimens [9,11,16,20,21,23,35,38-41]. The association between CD4^+ ^> 200 cells/mm^3 ^at baseline and the subsequent risk of developing DAIDS 3/4 TE while on treatment may have resulted from a bias. It is possible that the "healthier" patients with higher CD4^+ ^cell count at baseline remained on study longer than the sicker patients and therefore had more transaminase testing performed, and thus increasing the possibility of detection of elevated transaminases. In our study, patients with CD4^+ ^counts > 200 cell/mm^3 ^remained in the study nearly 3.5 months longer than patients with counts at or below 200 cells/mm^3^. The overall drug exposure difference is more pronounced when one considers treatment regimen; on the average TPV/r patients with CD4^+ ^counts above 200 remained in the study 8 month longer than their CPI/r treated counterparts (data not shown).

Inherent in many clinical trials are limitations in capturing all of the necessary risk factors associated with the outcome being evaluated. Although safety of patients was of paramount importance, the focus of these trials was on efficacy in a cohort of patients with highly advanced HIV disease and limited treatment options. As such, baseline information on known risk factors for hepatotoxicity, such as allergic predisposition, severe hepatic steatosis or hepatic cirrhosis [8] was obtained only through investigator and/or patient self-reporting; and therefore may have led to an underestimation of the presence of pre-existent liver disease, and hence an underestimation of the contribution of these to observed serious hepatic events associated with tipranavir in patients. In these trials, the baseline prevalence of any reported hepatic steatosis, cirrhosis or drug hypersensitivity was too low, 0.5% (6/1299), 0.2% (2/1299) and 1.9% (25/1299), respectively, to warrant evaluation of these important risk factors.

Another limitation to these analyses, found in HIV clinical studies in general, is the predominance of male patients. In the tipranavir Phase IIb and III clinical trials, only 10% of all patients were females, possibly limiting our ability to fully examine the role of gender on our study results and conclusions. While some researchers have shown a differential incidence of drug-related adverse events [42-44] based on gender, these results have not been confirmed by others [45,46].

In conclusion, TPV/r-based cART was associated with DAIDS Grade 3/4 ALT/AST elevations in treatment-experienced, HIV-1-infected patients with HIV advanced disease and limited treatment options; patients with elevated baseline ALT and/or AST levels and/or chronic viral hepatitis had the greatest risk. Of note, most of these transaminase elevations were not associated with clinically significant events; most patients with elevated transaminases experienced resolution without permanent discontinuation of TPV/r. Furthermore, Grade 3/4 ALT/AST elevations were not predictive of subsequent clinical hepatic events because the majority of patients remained asymptomatic. In this highly ARV-experienced patient population, the risk of DAIDS Grade 3/4 ALT/AST elevations should be balanced with the potential benefit of treatment with TPV/r in individuals for whom this represents an active ARV drug. Importantly, TPV/r demonstrated significantly greater virologic response than CPI/r in the RESIST trials, which enrolled more than 1400 treatment-experienced patients [6,30]. Further follow-up of these trials has indicated that TPV/r patients who achieve virologic suppression continue to do well through 156 weeks of treatment [31].

For HIV-positive patients being treated with TPV/r, healthcare providers should monitor patients for the emergence of clinically significant hepatic events. Appropriate laboratory and clinical monitoring should be conducted prior to initiating therapy with TPV/r, and throughout treatment. Increased frequency of monitoring should be considered when TPV/r is administered to patients with elevated baseline ALT/AST levels, or active HBV/HCV co-infection, as these patients may be at increased risk for developing further transaminase elevations.

## Competing interests

JM, PAR, MR and JOS are employees of Boehringer Ingelheim, the manufacturer of APTIVUS^® ^(tipranavir). The authors declare that they have no other competing interests.

## Authors' contributions

All authors participated in the data analysis and interpretation of results, as well as in revising the final manuscript.

## Pre-publication history

The pre-publication history for this paper can be accessed here:

http://www.biomedcentral.com/1471-2334/9/203/prepub
